# Dietary Diversity Changes and Cognitive Frailty in Chinese Older Adults: A Prospective Community-Based Cohort Study

**DOI:** 10.3390/nu15173784

**Published:** 2023-08-30

**Authors:** Wen-Fang Zhong, Wei-Qi Song, Xiao-Meng Wang, Zhi-Hao Li, Dong Shen, Dan Liu, Pei-Dong Zhang, Qiao-Qiao Shen, Fen Liang, Ying Nan, Jia-Xuan Xiang, Zi-Ting Chen, Chuan Li, Shi-Tian Li, Xiao-Gang Lv, Xiu-Rong Lin, Yue-Bin Lv, Xiang Gao, Virginia Byers Kraus, Xiao-Ming Shi, Chen Mao

**Affiliations:** 1 Department of Epidemiology, School of Public Health, Southern Medical University, Guangzhou 510515, China; zhongwf0613@hotmail.com (W.-F.Z.); songweiqi40@126.com (W.-Q.S.); wangxm0309@hotmail.com (X.-M.W.); zhihaoli2013@smu.edu.cn (Z.-H.L.); shendong@smu.edu.cn (D.S.); liudan0717@smu.edu.cn (D.L.); zpd911@gmail.com (P.-D.Z.); shenqiaoqiao0319@163.com (Q.-Q.S.); lflf9779@163.com (F.L.); ny357456@163.com (Y.N.); zitingchen2021@163.com (Z.-T.C.); lichuan202105@163.com (C.L.); lishitian6@163.com (S.-T.L.); lyuxiaox@163.com (X.-G.L.); uaenalllxr@163.com (X.-R.L.); 2 Department of Public Health and Preventive Medicine, School of Medicine, Jinan University, Guangzhou 510632, China; 3 Department of Neurosurgery, Nanfang Hospital, Southern Medical University, Guangzhou 510515, China; 4 School of Nursing, Southern Medical University, Guangzhou 510515, China; 5 National Institute of Environmental Health, Chinese Center for Disease Control and Prevention, Beijing 100021, China; lvyuebin@nieh.chinacdc.cn; 6 Department of Nutrition and Food Hygiene, School of Public Health, Institute of Nutrition, Fudan University, Shanghai 200433, China; xiang_gao@fudan.edu.cn; 7 Division of Rheumatology, Department of Medicine, Duke Molecular Physiology Institute, Duke University School of Medicine, Durham, NC 27701, USA; kraus004@duke.edu

**Keywords:** dietary diversity, cognitive frailty, older people, cohort study

## Abstract

Evidence for the effects of dietary diversity changes and cognitive frailty (CF) in the older adults is not clear. This study aimed to investigate the relationship between dietary diversity changes and CF in older adults Chinese. A total of 14,382 participants (mean age: 82.3 years) were enrolled. Dietary diversity scores (DDSs) were collected and calculated using a food frequency questionnaire. DDS changes between baseline and first follow-up were categorized into nine patterns. The associations between DDS changes and the incidence of CF were estimated using Cox proportional hazards models. During an 80,860 person-year follow-up, 3023 CF cases were identified. Groups with a decrease in DDS had increased CF risk compared with the high-to-high DDS group, with adjusted hazard ratios (HRs; 95% confidence intervals (Cis)) of 1.30 (1.06, 1.59), 2.04 (1.51, 2.74), and 1.81 (1.47, 2.22) for high-to-medium, high-to-low, and medium-to-low groups, respectively. Lower overall DDS groups were associated with greater CF risks, with HRs (95% CIs) of 1.49 (1.19, 1.86) for the low-to-medium group and 1.96 (1.53, 2.52) for the low-to-low group. Compared with the high-to-high group, significant associations with CF were found in other DDS change groups; HRs ranged from 1.38 to 3.12 for the plant-based DDS group and from 1.24 to 1.32 for the animal-based DDS group. Additionally, extreme and moderate declines in overall DDS increased CF risk compared with stable DDS, with HRs (95% CIs) of 1.67 (1.50, 1.86) and 1.13 (1.03, 1.24), respectively. In conclusion, among older adults, a declining or persistently low DDS and a moderately or extremely declining DDS were linked to higher incident CF. Plant-based DDS changes correlated more strongly with CF than animal-based DDS changes.

## 1. Introduction

The rising prevalence of age-related diseases among the older adults has garnered increasing attention due to the aging of the global population. Cognitive frailty (CF) is a clinical symptom combining cognitive impairment and physical frailty [1], reflecting a state of nonspecific vulnerability. Previous studies have investigated whether CF may increase the risk of adverse outcomes [2,3] and mortality [4,5] in comparison with physical frailty or cognitive impairment alone [6,7,8]. Importantly, CF can be reversible if effective interventions are employed [9]. Therefore, it is imperative to identify modifiable risk factors and implement preventive and health promotion strategies in the early or reversible stages.

In recent years, the modifiable risk factor of overall diet quality has received plenty of attention, with consideration given to the effects of various nutrients and other bioactive constituents [10,11,12]. The dietary diversity score (DDS) is recognized as a valid and clinically pertinent measure that reflects nutrient sufficiency and diet quality among the numerous methods used to evaluate overall diet. Some studies have found a correlation or interaction between DDS and nutritional status [13,14], suggesting that a higher DDS may be indicative of nutrient sufficiency and improved health in older adults. Furthermore, the DDS has been shown to be related to the risk of numerous diseases [15,16]. Cross-sectional research has suggested that CF deterioration was associated with a lower DDS [17], but its design limited any inference of a causal relationship. Moreover, the above study only measured baseline DDS; ignoring measurement bias could alter the results with the evolution of diet during follow-up. Dietary diversity changes have been confirmed to be linked to cognitive impairment [18] and mortality [19] in the older adults in previous longitudinal studies. However, the association between changes from baseline DDS to follow-up DDS and CF has yet to be fully explored.

To fill this knowledge gap, we explored DDS change patterns and CF on the basis of a prospective cohort study of the Chinese Longitudinal Healthy Longevity Survey (CLHLS).

## 2. Materials and Methods

### 2.1. Study Setting and Participants

This study included participants in the CLHLS. The CLHLS commenced in 1998 and enrolled new participants during every follow-up to maintain the sample size. It covers nearly 85% of the population across 23 provinces in China. Data were obtained through a structured questionnaire through face-to-face interviews; the details regarding the study design were reported earlier [20].

From the CLHLS, we analyzed three successive 9-year cohorts of longitudinal data (the 2002, 2005, and 2008 cohorts); each consisting of four waves of data (participants recruited in the 2002 cohort were followed up in the 2005, 2008, and 2011 waves; those newly recruited in the 2005 cohort were followed up in the 2008, 2011, and 2014 waves; and those newly recruited in the 2008 cohort were followed up in the 2011, 2014, and 2017 waves. Of the initial 48,656 individuals who participated in our study, 26,533 participants were excluded: 460 with an age of <65 years, 15 with incomplete dietary data at baseline, 24,425 with incomplete dietary data at the first follow-up, 1596 with CF at baseline, and 15 who were lost to the follow-up CF assessment. Among the remaining 22,123 participants, we excluded those duplicated in three cohorts (*n* = 7741), resulting in a final sample size of 14,382 (Figure S1).

### 2.2. Definitions of DDS Change Patterns

The valid and reliable simplified food-frequency questionnaire was used to collect the frequency of consumption of the following 9 food items [21]: fresh vegetables, fruit, tea, garlic, beans, preserved vegetables, meat, fish, and eggs. We calculated the DDS according to the method proposed by Kant [22] and considered both the number and frequency of food categories. In general, we constructed an overall DDS (a score of 0 to 18), a plant-based DDS (a score of 0 to 12), and an animal-based DDS (a score of 0 to 6). Subsequently, we divided participants into 3 groups by the trisection of the total scores (high, medium, and low) and combined both baseline and the first follow-up visit DDS to form 9 relative DDS change patterns. We also computed the absolute change scores (the numerical value of the first follow-up DDS minus the baseline DDS) and categorized participants into 5 absolute DDS change patterns [19]. More details are provided in the supplementary methods [19,23,24,25,26,27].

### 2.3. Assessment of CF

Cognition impairment was defined by combining education with the scores of the Chinese version of the Mini-Mental State Examination. Physical frailty was evaluated using the modified Fried criteria. The present study defined CF as the co-occurrence of cognitive impairment and physical frailty [1] (details can be found in the supplementary methods). The follow-up period was from enrolment until the end of follow-up or incident CF, whichever occurred first.

### 2.4. Assessment of Covariates

We included explanatory variable groups based on previous studies [18,19]. Potential confounders included demographics, lifestyle, and health status. Demographics were age (continuous), sex, living areas, marital status, occupation, years of education, income source, and sufficient income. Lifestyle included living arrangement, body mass index (BMI; continuous), regular exercise, smoking status, and drinking status. Health status included 4 chronic diseases: hypertension, diabetes, stroke, and heart disease.

### 2.5. Statistical Analysis

The missing data rates for all variables were less than 1.31% (Table S1). Multiple imputations were used to impute missing covariates in order to reduce inferential bias and maximize statistical power [28]. The Cox proportional hazards regression model was used to calculate the hazard ratios (HRs) and 95% confidence intervals (CIs) for DDS change patterns and CF risk. 

We tested the assumption of the proportional risk and found that it was satisfied. Two sets of models were conducted. The base model controlled only for age and sex; the fully adjusted model further controlled for living area, marital status, occupation, years of education, income source, sufficient income, living arrangement, BMI, regular exercise, smoking status, drinking status, hypertension, diabetes, stroke, and heart disease.

Subgroup analyses were conducted according to age, sex, living areas, smoking status, drinking status, and regular exercise to examine the association between DDS change patterns and CF. To evaluate the robustness of the findings, multiple sensitivity analyses were performed, including further adjustment for the number of teeth and the usage of dentures, additional adjustment for the year of recruitment, the exclusion of participants with dementia at baseline, and the exclusion of participants with missing covariates. Analyses were all performed using R 4.2.2. with a two-sided *p*-value of <0.05 indicating statistical significance.

## 3. Results

### 3.1. Baseline Characteristics

A total of 14,382 participants (53.7% women; mean age: 82.3 years) were included in the present study. According to relative DDS change patterns, the low-to-high group had the fewest participants (117, 0.8%), while the medium-to-medium group had the most participants (6939, 48.2%). Participants in the high-to-high pattern group were significantly younger and were more likely to be male, to live in urban areas, to be married, no not be a farmer, to have longer years of education, to have a pension and sufficient income, to live with a family member, to have a higher BMI, to regularly exercise, to be a current or former smoker, to be a current or former drinker, and to have hypertension, diabetes, and heart disease (Table 1). 

### 3.2. Relative DDS Change Patterns and CF

**Figure 1:**
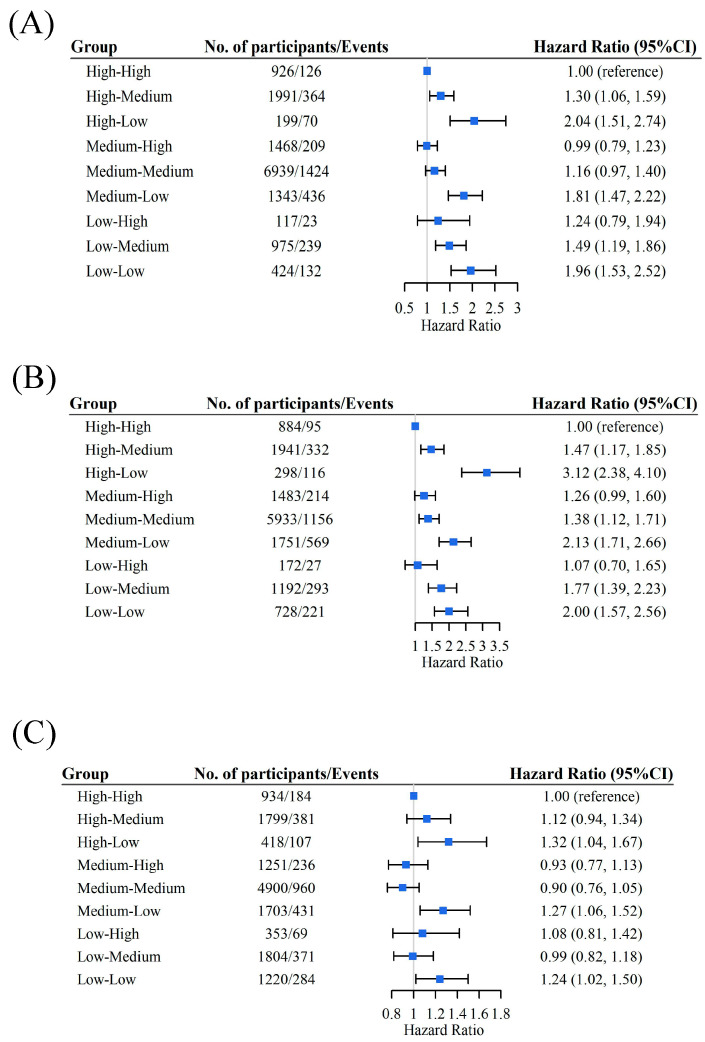
Association between change patterns of overall DDS (**A**), plant-based DDS (**B**), and animal-based DDS (**C**) and CF. Adjusted for age, sex, living area, marital status, occupation, years of education, income source, sufficient income, living arrangement, BMI, regular exercise, smoking status, drinking status, hypertension, diabetes, stroke, and heart disease. The blue boxes in the figure represent the hazard ratios, and the horizontal lines indicate the corresponding 95% confidence intervals.

During the 80,860 person-years of follow-up (with a median follow-up time of 5.5 years), 3023 incident CF cases were recorded. The incidence of CF was lowest in the high-to-high group (23.9 per 1000 person-years), while it was greatest in the high-to-low group (71.4 per 1000 person-years). Compared with participants in the high-to-high group, a significantly increased risk of CF was observed in participants in the groups with a decrease in DDS. Specifically, regarding overall DDS, the adjusted HRs (95%CI) of developing CF were 1.30 (1.06, 1.59) for the high-to-medium group, 2.04 (1.51, 2.74) for the high-to-low group, and 1.81 (1.47, 2.22) for the medium-to-low group. Lower overall DDS patterns also increased CF risk with HRs of 1.49 (1.19, 1.86) for the low-to-medium group and 1.96 (1.53, 2.52) for the low-to-low group (Figure 1A).

Similar relationships were observed between plant-based and animal-based DDSs and CF. Compared with the high-to-high group, significant HRs related to plant-based DDS ranged from 1.38 to 3.12 for the different plant food groups, and significant HRs related to animal-based DDS ranged from 1.24 to 1.32 for the different animal food groups. In particular, compared with the high-to-high group, the HR estimates for the plant-based DDS were 1.47 (1.17, 1.85) in the high-to-medium group, 3.12 (2.38, 4.10) in the high-to-low group, 1.38 (1.12, 1.71) in the medium-to-medium group, 2.13 (1.71, 2.66) in the medium-to-low group, 1.77 (1.39, 2.23) in the low-to-medium group, and 2.00 (1.57, 2.56) in the low-to-low group (Figure 1B). For the animal-based DDS, the HR estimates were 1.32 (1.04, 1.67) in the high-to-low group, 1.27 (1.06, 1.52) in the medium-to-low group, and 1.24 (1.02, 1.50) in the low-to-low group (Figure 1C). Table 2 demonstrates the relationship between numerous food items and CF. These results were consistent with the main findings.

Subgroup analyses (Table 3) showed a significant interaction between DDS change and sex on CF. Specifically, males were more susceptible to the effects of DDS reduction on CF risk (*p* for interaction = 0.020). Additionally, a significant interaction was observed between DDS change and CF stratified by smoking status (*p* for interaction = 0.026). When stratified by age, living area, drinking, and regular exercise, the associations resembled our major findings. When we further adjusted for the number of teeth, usage of dentures, and the year of recruitment; excluded participants with dementia at baseline; or excluded participants with missing covariates, the sensitivity analyses indicated no substantial changes (Table S3).

### 3.3. Absolute DDS Change Patterns and CF

**Figure 2:**
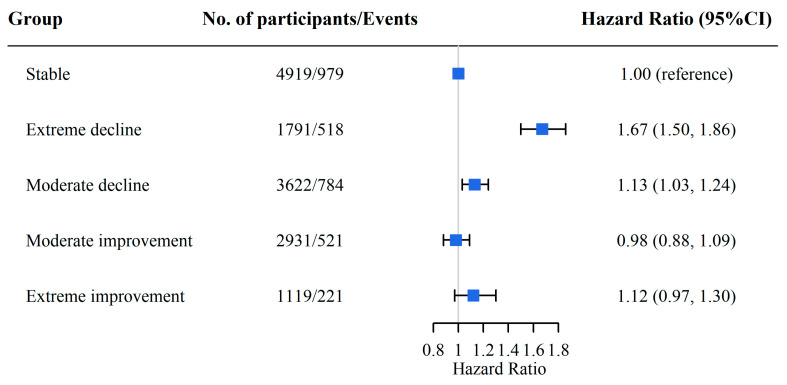
The association between absolute DDS change groups and CF. Adjusted for age, sex, living area, marital status, occupation, years of education, income source, sufficient income, living arrangement, BMI, regular exercise, smoking status, drinking status, hypertension, diabetes, stroke, and heart disease. The blue boxes in the figure represent the hazard ratios, and the horizontal lines indicate the corresponding 95% confidence intervals.

We regarded a DDS that remained stable as a reference and found that a declining DDS was associated with a higher risk of CF. Specifically, the HRs (95% CI) were 1.67 (1.50, 1.86) for the extreme decline group and 1.13 (1.03, 1.24) for the moderate decline group, whereas the moderate and extreme improvement groups did not have a significantly increased risk of CF (*p* > 0.05) (Figure 2). Similar relationships were observed between plant-based or animal-based absolute DDS change patterns and CF (Table S4) and in the subgroup analyses (Table S5).

## 4. Discussion

In this community-based prospective study involving 14,382 Chinese adults aged 65 years and older, participants with the highest DDS had the lowest incidence rates of CF. We found that a consistently higher DDS was related to a lower CF risk, while DDSs that declined extremely or remained low were related to a higher CF risk. We provided evidence that dynamic changes in DDS, as a flexible intervention strategy, have potential benefits among the older adults. These results highlight the importance of higher dietary diversity for CF prevention.

Dietary diversity has been identified as beneficial for reductions in cognitive impairment [29] and mortality risk [30]. However, the relationship between DDS changes and the incident CF remains unknown. In this study, a higher DDS was related to decreased CF risk, consistent with previous investigations of various dietary quality indices, such as the Mediterranean diet, the Healthy Eating Index 2015, the Mediterranean–DASH Intervention for Neurodegenerative Delay, and the Mediterranean Dietary Approach to Systolic Hypertension diet [31,32,33]. Given the challenges of assessing overall dietary quality with the measurement of the abovementioned indices in a clinical setting, the DDS emerges as a more practical approach without quantitative measurements. Our findings emphasize the significance of maintaining a high DDS, which may have valuable public health implications for older adults.

Global dietary guidelines recommend a varied diet [34,35,36], although specifics may vary by the study population and country. A monotonous diet devoid of a variety of carbohydrate foods, fruits, vegetables, and animal foods may not provide all the necessary nutrients [37]. Low dietary diversity scores may increase the likelihood of nutritional deficiencies, particularly in older adults [38]. Both declining and persistently low plant-based and animal-based DDSs were significantly associated with an increased risk of CF, whereas the association was stronger for plant-based DDS changes. In addition, consuming more fresh vegetables, fruit, tea, garlic, and meat was associated with lower incident CF. Previous studies have indicated that an increase in the consumption of these nutrients can reduce the incidence of cognitive impairment and physical frailty [39,40,41,42]. Several plausible mechanisms could explain this relationship. For instance, fresh vegetables, fruit, tea, and garlic are regarded as good sources of antioxidants to reduce inflammation and oxidative stress, which mitigate the loss of muscle and bone mass and the degeneration of central nervous system function [43]. Meat contains high-quality protein, which is beneficial for reversing or preventing age-related muscle tissue wasting and cognitive dysfunction [44].

Our findings indicate that maintaining a stable, high DDS can significantly reduce CF risk. This is partially consistent with the results of the Rotterdam study [45], which found that adherence to a healthy dietary pattern resulted in less physical frailty over time. It is noteworthy that the interpretation of DDS for specific foods was consistent with the overall DDS, incorporating a simultaneous analysis of all nine dietary categories. For example, in our study, when fresh vegetable consumption dropped from “often-to-often” to “often-to-occasionally”, the incidence of CF increased by 27.5 per 1000 person-years, indicating that even a slight reduction in the frequency at which older individuals consume vegetables can have negative consequences.

Another interesting finding was that participants with an extreme decline or moderate decline in DDS had a higher CF risk than those with stable DDSs. Our investigation regarding the characteristics of participants with an extreme decline or moderate decline in DDS reveals that the higher risk may be associated with inferior familial care (such as not being married due to being single, divorced, or bereaved), and a higher prevalence of chronic diseases (such as hypertension) (Table S2). Surprisingly, those in the high-to-high group were mainly smokers and drinkers (Table 1); this finding exhibited contrary characteristics to our initial expectations and is presumed to be associated with living in urban areas or receiving better family care.

The strengths of this study include the prospective design, large sample size, and the evaluation of dynamic changes in diet quality. However, several limitations should be considered. First, the research did not record quantitative food consumption data; therefore, energy intake was not accounted for. Second, self-reported data to calculate DDS may have introduced recall bias. Third, given the observational nature of the study, residual confounding and reverse causality may exist. Finally, caution should be exercised when generalizing these findings to other populations or countries, as the study was conducted among Chinese older adults.

## 5. Conclusions

A declining or persistently low DDS and a moderately or extremely declining DDS were associated with a significantly higher incidence of CF risk among Chinese adults aged 65 years and older. Notably, the relationship between plant-based DDS changes and CF was stronger than that between animal-based DDS changes and CF. This is the first study, to our knowledge, to provide evidence of the association between changes in DDS and CF among the Chinese older adults population. Our results emphasize the importance of dynamic changes in dietary diversity for the prevention of CF and improved health among older adults, providing a practical message to this population.

## Figures and Tables

**Table 1:** Baseline characteristics of older adults according to DDS change patterns.

Characteristics ^a^	Total	DDS Change Patterns from Baseline to First Follow-Up								
		High to High	High to Medium	High to Low	Medium to High	Medium to Medium	Medium to Low	Low to High	Low to Medium	Low to Low
No. of participants	14,382	926	1991	199	1468	6939	1343	117	975	424
Age, mean (SD)	82.3 (10.8)	79.1 (10.6)	81.2 (10.6)	85.2 (11.2)	80.3 (10.3)	82.5 (10.9)	84.7 (10.9)	84.4 (11.7)	84.0 (10.5)	85.5 (9.9)
Sex										
Female	7729 (53.7)	356 (38.4)	913 (45.9)	115 (57.8)	662 (45.1)	3829 (55.2)	864 (64.3)	77 (65.8)	620 (63.6)	293 (69.1)
Male	6653 (46.3)	570 (61.6)	1078 (54.1)	84 (42.2)	806 (54.9)	3110 (44.8)	479 (35.7)	40 (34.2)	355 (36.4)	131 (30.9)
Living area										
Rural	8431 (58.6)	343 (37.0)	959 (48.2)	123 (61.8)	708 (48.2)	4269 (61.5)	944 (70.3)	69 (59.0)	704 (72.2)	312 (73.6)
Urban	5951 (41.4)	583 (63.0)	1032 (51.8)	76 (38.2)	760 (51.8)	2670 (38.5)	399 (29.7)	48 (41.0)	271 (27.8)	112 (26.4)
Marital status										
Married	6086 (42.3)	541 (58.4)	977 (49.1)	64 (32.2)	721 (49.1)	2836 (40.9)	456 (34.0)	37 (31.6)	328 (33.6)	126 (29.7)
Not married	8296 (57.7)	385 (41.6)	1014 (50.9)	135 (67.8)	747 (50.9)	4103 (59.1)	887 (66.0)	80 (68.4)	647 (66.4)	298 (70.3)
Occupation										
Farmer	9111 (63.4)	327 (35.3)	1054 (52.9)	126 (63.3)	756 (51.5)	4713 (67.9)	989 (73.6)	78 (66.7)	743 (76.2)	325 (76.7)
Other	5271 (36.6)	599 (64.7)	937 (47.1)	73 (36.7)	712 (48.5)	2226 (32.1)	354 (26.4)	39 (33.3)	232 (23.8)	99 (23.3)
Years of education, y										
0	8271 (57.5)	321 (34.7)	952 (47.8)	118 (59.3)	672 (45.8)	4145 (59.7)	942 (70.1)	87 (74.4)	710 (72.8)	324 (76.4)
≥1	6111 (42.5)	605 (65.3)	1039 (52.2)	81 (40.7)	796 (54.2)	2794 (40.3)	401 (29.9)	30 (25.6)	265 (27.2)	100 (23.6)
Income source										
Pension	2930 (20.4)	457 (49.4)	597 (30.0)	30 (15.1)	453 (30.9)	1140 (16.4)	124 (9.2)	17 (14.5)	89 (9.1)	23 (5.4)
Others	11,452 (79.6)	469 (50.6)	1394 (70.0)	169 (84.9)	1015 (69.1)	5799 (83.6)	1219 (90.8)	100 (85.5)	886 (90.9)	401 (94.6)
Sufficient income										
Yes	11,395 (79.2)	856 (92.4)	1775 (89.2)	170 (85.4)	1238 (84.3)	5485 (79.0)	978 (72.8)	84 (71.8)	575 (59.0)	234 (55.2)
No	2987 (20.8)	70 (7.6)	216 (10.8)	29 (14.6)	230 (15.7)	1454 (21.0)	365 (27.2)	33 (28.2)	400 (41.0)	190 (44.8)
Living arrangement										
Living alone/at nursing home	2533 (17.6)	99 (10.7)	257 (12.9)	37 (18.6)	200 (13.6)	1224 (17.6)	280 (20.8)	24 (20.5)	270 (27.7)	142 (33.5)
Living with family member	11,849 (82.4)	827 (89.3)	1734 (87.1)	162 (81.4)	1268 (86.4)	5715 (82.4)	1063 (79.2)	93 (79.5)	705 (72.3)	282 (66.5)
BMI, kg/m^2^	20.23 (4.3)	21.7 (4.2)	20.9 (4.5)	19.9 (3.6)	20.8 (5.3)	20.1 (4.3)	19.5 (3.6)	19.7 (3.8)	19.3 (3.5)	18.8 (3.7)
Regular exercise	5215 (36.3)	536 (57.9)	931 (46.8)	90 (45.2)	633 (43.1)	2291 (33.0)	375 (27.9)	24 (20.5)	242 (24.8)	93 (21.9)
Smoking status										
Current smoker	3114 (21.7)	247 (26.7)	500 (25.1)	51 (25.6)	367 (25.0)	1459 (21.0)	239 (17.8)	24 (20.5)	178 (18.3)	49 (11.6)
Former smoker	2073 (14.4)	184 (19.9)	337 (16.9)	25 (12.6)	253 (17.2)	945 (13.6)	154 (11.5)	17 (14.5)	107 (11.0)	51 (12.0)
Nonsmoker	9195 (63.9)	495 (53.5)	1154 (58.0)	123 (61.8)	848 (57.8)	4535 (65.4)	950 (70.7)	76 (65.0)	690 (70.8)	324 (76.4)
Drinking status										
Current drinker	3229 (22.5)	308 (33.3)	538 (27.0)	54 (27.1)	374 (25.5)	1496 (21.6)	238 (17.7)	20 (17.1)	155 (15.9)	46 (10.8)
Former drinker	1551 (10.8)	105 (11.3)	231 (11.6)	24 (12.1)	185 (12.6)	710 (10.2)	125 (9.3)	18 (15.4)	114 (11.7)	39 (9.2)
Nondrinker	9602 (66.8)	513 (55.4)	1222 (61.4)	121 (60.8)	909 (61.9)	4733 (68.2)	980 (73.0)	79 (67.5)	706 (72.4)	339 (80.0)
Hypertension	6503 (45.2)	464 (50.1)	930 (46.7)	97 (48.7)	703 (47.9)	2994 (43.1)	584 (43.5)	66 (56.4)	457 (46.9)	208 (49.1)
Diabetes	322 (2.2)	25 (2.7)	60 (3.0)	2 (1.0)	35 (2.4)	159 (2.3)	21 (1.6)	2 (1.7)	15 (1.5)	3 (0.7)
Stroke	668 (4.6)	53 (5.7)	112 (5.6)	8 (4.0)	80 (5.4)	290 (4.2)	57 (4.2)	7 (6.0)	41 (4.2)	20 (4.7)
Heart disease	1273 (8.9)	121 (13.1)	191 (9.6)	16 (8.0)	163 (11.1)	545 (7.9)	106 (7.9)	10 (8.5)	77 (7.9)	44 (10.4)

^a^ Values represent the mean ± SD or number (percentage). BMI: body mass index; DDS: dietary diversity score; SD: standard deviation.

**Table 2:** The association between DDS change patterns and CF in nine foods.

Foods	DDS Change Patterns from Baseline to First Follow-Up								
	Often to Often	Often to Occasionally	Often to Rarely	Occasionally to Often	Occasionally to Occasionally	Occasionally to Rarely	Rarely to Often	Rarely to Occasionally	Rarely to Rarely
Fresh vegetables									
No. of CF/person-years	2153/65,525	330/5468	182/1693	197/5247	48/1107	36/422	51/1033	17/241	9/124
Incidence rate of CF	32.9	60.4	107.5	37.5	43.4	85.3	49.4	70.5	72.6
HR (95%CI) (model 1) ^a^	1.00 (ref)	1.62 (1.44, 1.82)	2.42 (2.08, 2.82)	1.04 (0.90, 1.20)	1.04 (0.78, 1.39)	2.53 (1.82, 3.51)	1.08 (0.82, 1.43)	1.90 (1.18, 3.07)	1.53 (0.79, 2.94)
HR (95%CI) (model 2) ^b^	1.00 (ref)	1.61 (1.43, 1.81)	2.37 (2.03, 2.76)	1.04 (0.90, 1.21)	1.02 (0.76, 1.36)	2.47 (1.77, 3.43)	1.09 (0.83, 1.44)	1.76 (1.09, 2.85)	1.57 (0.82, 3.03)
Fresh fruit									
No. of CF/person-years	488/16,179	312/8991	263/4986	297/9610	451/13,593	411/8632	185/4311	266/7064	350/7495
Incidence rate of CF	30.2	34.7	52.7	30.9	33.2	47.6	42.9	37.7	46.7
HR (95%CI) (model 1) ^a^	1.00 (ref)	1.08 (0.93, 1.24)	1.51 (1.30, 1.76)	1.02 (0.89, 1.18)	1.05 (0.92, 1.19)	1.39 (1.22, 1.59)	1.32 (1.12, 1.57)	1.20 (1.03, 1.40)	1.38 (1.20, 1.58)
HR (95%CI) (model 2) ^b^	1.00 (ref)	1.11 (0.96, 1.28)	1.59 (1.36, 1.85)	1.08 (0.93, 1.25)	1.14 (1.00, 1.30)	1.52 (1.32, 1.74)	1.38 (1.16, 1.64)	1.30 (1.12, 1.52)	1.49 (1.29, 1.72)
Tea									
No. of CF/person-years	293/13,491	164/4813	354/8405	110/4439	105/3175	299/6852	191/5836	240/6003	1267/27,846
Incidence rate of CF	21.7	34.1	42.1	24.8	33.1	43.6	32.7	40.0	45.5
HR (95%CI) (model 1) ^a^	1.00 (ref)	1.42 (1.18, 1.72)	1.47 (1.26, 1.72)	1.12 (0.90, 1.40)	1.29 (1.03, 1.62)	1.39 (1.18, 1.64)	1.26 (1.05, 1.51)	1.36 (1.15, 1.62)	1.40 (1.23, 1.60)
HR (95%CI) (model 2) ^b^	1.00 (ref)	1.46 (1.20, 1.76)	1.51 (1.29, 1.76)	1.08 (0.86, 1.34)	1.26 (1.00, 1.57)	1.41 (1.20, 1.66)	1.22 (1.01, 1.47)	1.37 (1.16, 1.63)	1.43 (1.25, 1.63)
Garlic									
No. of CF/person-years	120/5695	257/8261	179/3787	240/8283	608/19,050	544/11,829	111/3388	404/10,050	560/10,517
Incidence rate of CF	21.1	31.1	47.3	29.0	31.9	46.0	32.8	40.2	53.2
HR (95%CI) (model 1) ^a^	1.00 (ref)	1.28 (1.03, 1.59)	1.71 (1.35, 2.15)	1.23 (0.99, 1.53)	1.15 (0.94, 1.39)	1.54 (1.27, 1.88)	1.32 (1.02, 1.71)	1.34 (1.09, 1.64)	1.63 (1.33, 1.98)
HR (95%CI) (model 2) ^b^	1.00 (ref)	1.33 (1.07, 1.65)	1.79 (1.42, 2.26)	1.24 (0.99, 1.54)	1.20 (0.98, 1.46)	1.60 (1.31, 1.95)	1.31 (1.01, 1.69)	1.38 (1.13, 1.70)	1.66 (1.36, 2.02)
Beans									
No. of CF/person-years	314/9510	553/13,234	139/2863	307/8826	1019/29,672	251/6107	85/1848	239/6256	116/2544
Incidence rate of CF	33.0	41.8	48.6	34.8	34.3	41.1	46	38.2	45.6
HR (95%CI) (model 1) ^a^	1.00 (ref)	0.95 (0.83, 1.09)	1.21 (0.99, 1.48)	1.00 (0.85, 1.17)	0.79 (0.69, 0.89)	0.91 (0.77, 1.08)	1.23 (0.97, 1.56)	0.92 (0.78, 1.09)	1.07 (0.87, 1.33)
HR (95%CI) (model 2) ^b^	1.00 (ref)	0.99 (0.86, 1.14)	1.30 (1.06, 1.59)	1.01 (0.86, 1.18)	0.84 (0.74, 0.96)	0.99 (0.84, 1.18)	1.24 (0.97, 1.57)	0.98 (0.83, 1.17)	1.15 (0.93, 1.43)
Preserved vegetables									
No. of CF/person-years	196/6850	240/8209	281/6280	150/5892	392/12,651	480/11,442	133/3713	379/10,198	772/15,625
Incidence rate of CF	28.6	29.2	44.7	25.5	31.0	42.0	35.8	37.2	49.4
HR (95%CI) (model 1) ^a^	1.00 (ref)	0.91 (0.75, 1.10)	1.25 (1.04, 1.50)	0.79 (0.63, 0.97)	0.85 (0.71, 1.01)	0.95 (0.80, 1.12)	0.89 (0.71, 1.11)	0.92 (0.77, 1.09)	1.06 (0.90, 1.24)
HR (95%CI) (model 2) ^b^	1.00 (ref)	0.94 (0.77, 1.13)	1.26 (1.05, 1.52)	0.79 (0.64, 0.98)	0.86 (0.72, 1.02)	0.97 (0.82, 1.15)	0.89 (0.71, 1.11)	0.93 (0.79, 1.11)	1.06 (0.90, 1.24)
Meat									
No. of CF/person-years	557/14,800	454/11,182	111/2037	387/10,721	769/25,032	263/5612	71/1891	228/6138	183/3446
Incidence rate of CF	37.6	40.6	54.5	36.1	30.7	46.9	37.5	37.1	53.1
HR (95%CI) (model 1) ^a^	1.00 (ref)	1.14 (1.01, 1.30)	1.57 (1.28, 1.92)	1.00 (0.88, 1.14)	0.91 (0.81, 1.01)	1.56 (1.35, 1.81)	1.18 (0.92, 1.51)	1.07 (0.92, 1.25)	1.64 (1.38, 1.93)
HR (95%CI) (model 2) ^b^	1.00 (ref)	1.14 (1.01, 1.29)	1.60 (1.30, 1.97)	1.02 (0.89, 1.16)	0.93 (0.83, 1.04)	1.57 (1.35, 1.83)	1.11 (0.87, 1.43)	1.09 (0.93, 1.27)	1.62 (1.36, 1.92)
Fish									
No. of CF/person-years	135/3865	319/8060	97/1900	173/5120	969/32,005	470/9746	54/1443	407/10,164	399/8556
Incidence rate of CF	34.9	39.6	51.1	33.8	30.3	48.2	37.4	40.0	46.6
HR (95%CI) (model 1) ^a^	1.00 (ref)	1.07 (0.88, 1.31)	1.23 (0.95, 1.59)	0.90 (0.71, 1.12)	0.84 (0.70, 1.01)	1.17 (0.96, 1.42)	0.99 (0.72, 1.36)	0.95 (0.78, 1.16)	1.16 (0.95, 1.40)
HR (95%CI) (model 2) ^b^	1.00 (ref)	1.10 (0.90, 1.35)	1.32 (1.02, 1.72)	0.91 (0.73, 1.14)	0.89 (0.74, 1.07)	1.25 (1.03, 1.52)	1.03 (0.75, 1.42)	1.03 (0.85, 1.26)	1.25 (1.02, 1.52)
Eggs									
No. of CF/person-years	659/15,901	527/13,172	116/2414	389/10,561	724/23,000	216/5418	95/2376	199/5296	98/2721
Incidence rate of CF	41.4	40.0	48.1	36.8	31.5	39.9	40.0	37.6	36.0
HR (95%CI) (model 1) ^a^	1.00 (ref)	0.93 (0.83, 1.04)	1.37 (1.12, 1.67)	0.91 (0.80, 1.03)	0.76 (0.68, 0.84)	0.96 (0.82, 1.12)	1.04 (0.84, 1.28)	0.93 (0.79, 1.09)	1.01 (0.82, 1.25)
HR (95%CI) (model 2) ^b^	1.00 (ref)	0.96 (0.85, 1.07)	1.42 (1.16, 1.73)	0.92 (0.81, 1.05)	0.79 (0.71, 0.88)	1.02 (0.87, 1.20)	1.02 (0.82, 1.26)	0.95 (0.81, 1.12)	1.04 (0.84, 1.29)

Incidence rate of CF (1000 person-years). ^a^ Adjusted for age and sex. ^b^ Adjusted for model 1 plus living area, marital status, occupation, years of education, income source, sufficient income, living arrangement, BMI, regular exercise, smoking status, drinking status, hypertension, diabetes, stroke, and heart disease.

**Table 3:** The associations between DDS change patterns and CF in subgroups.

Subgroups	DDS Change Patterns from Baseline to First Follow-Up									*p* for Interaction
	High to High	High to Medium	High to Low	Medium to High	Medium to Medium	Medium to Low	Low to High	Low to Medium	Low to Low	
Age, years										0.221
<80	1.00 (ref)	0.99 (0.57, 1.74)	2.35 (0.92, 5.96)	1.28 (0.74, 2.20)	1.34 (0.82, 2.17)	2.04 (1.17, 3.59)	0.65 (0.09, 4.91)	1.75 (0.95, 3.22)	1.84 (0.85, 3.98)	
≥80	1.00 (ref)	1.31 (1.05, 1.63)	2.00 (1.46, 2.73)	0.91 (0.71, 1.16)	1.12 (0.91, 1.37)	1.75 (1.41, 2.18)	1.24 (0.78, 1.97)	1.41 (1.11, 1.79)	1.90 (1.45, 2.49)	
Sex										0.020
Female	1.00 (ref)	0.99 (0.77, 1.28)	1.37 (0.96, 1.98)	0.74 (0.56, 0.99)	0.85 (0.68, 1.08)	1.30 (1.02, 1.67)	0.97 (0.59, 1.60)	1.02 (0.78, 1.34)	1.44 (1.07, 1.95)	
Male	1.00 (ref)	1.82 (1.30, 2.57)	3.76 (2.27, 6.23)	1.50 (1.04, 2.17)	1.83 (1.33, 2.51)	2.99 (2.09, 4.27)	1.43 (0.51, 3.99)	2.89 (1.95, 4.30)	3.14 (1.95, 5.05)	
Living area										0.789
Rural	1.00 (ref)	1.22 (0.89, 1.69)	1.93 (1.27, 2.92)	1.02 (0.72, 1.44)	1.09 (0.82, 1.46)	1.67 (1.23, 2.27)	1.34 (0.75, 2.39)	1.37 (0.99, 1.90)	1.98 (1.39, 2.81)	
Urban	1.00 (ref)	1.34 (1.02, 1.74)	2.09 (1.35, 3.23)	0.95 (0.71, 1.28)	1.21 (0.95, 1.55)	1.97 (1.47, 2.63)	1.00 (0.48, 2.08)	1.63 (1.17, 2.29)	1.71 (1.13, 2.58)	
Smoking										0.026
Never smoker	1.00 (ref)	1.16 (0.91, 1.48)	2.10 (1.48, 2.96)	0.88 (0.67, 1.15)	1.00 (0.80, 1.24)	1.46 (1.14, 1.86)	1.04 (0.62, 1.76)	1.22 (0.94, 1.59)	1.61 (1.20, 2.15)	
Current or former smoker	1.00 (ref)	1.57 (1.08, 2.28)	1.75 (0.99, 3.12)	1.22 (0.81, 1.83)	1.61 (1.14, 2.27)	3.03 (2.07, 4.45)	1.81 (0.76, 4.33)	2.33 (1.52, 3.57)	3.28 (1.97, 5.45)	
Drinking										0.145
Never drinker	1.00 (ref)	1.31 (1.01, 1.70)	1.94 (1.31, 2.87)	0.98 (0.74, 1.30)	1.16 (0.92, 1.48)	1.75 (1.35, 2.26)	1.55 (0.92, 2.60)	1.45 (1.10, 1.92)	1.77 (1.31, 2.40)	
Current or former drinker	1.00 (ref)	1.26 (0.90, 1.76)	2.16 (1.37, 3.41)	1.03 (0.72, 1.47)	1.17 (0.86, 1.58)	1.99 (1.41, 2.81)	0.72 (0.28, 1.81)	1.58 (1.07, 2.32)	3.05 (1.89, 4.90)	
Regular exercises										0.179
Yes	1.00 (ref)	1.16 (0.86, 1.56)	1.45 (0.91, 2.31)	0.95 (0.69, 1.32)	1.16 (0.89, 1.51)	1.59 (1.16, 2.18)	0.82 (0.20, 3.34)	1.15 (0.78, 1.70)	1.58 (0.96, 2.60)	
No	1.00 (ref)	1.39 (1.05, 1.85)	2.61 (1.77, 3.87)	1.01 (0.74, 1.38)	1.18 (0.91, 1.54)	1.90 (1.44, 2.52)	1.33 (0.81, 2.19)	1.64 (1.22, 2.20)	2.11 (1.53, 2.91)	

Adjusted for age, sex, living area, marital status, occupation, years of education, income source, sufficient income, living arrangement, BMI, regular exercise, smoking status, drinking status, hypertension, diabetes, stroke, and heart disease.

## Data Availability

Available from the Peking University on request (https://opendata.pku.edu.cn; accessed on 3 April 2020).

## Supplementary Materials

The following supporting information can be downloaded at: https://www.mdpi.com/article/10.3390/nu15173784/s1, Figure S1. Flowchart of participant enrollment. Table S1. The numbers (percentages) of participants with missing covariates. Table S2. Baseline characteristics of older adults according to absolute DDS change patterns. Table S3. Sensitivity analyses of the association between DDS change patterns and CF. Table S4. The association between absolute DDS change groups and CF in plant-based and animal-based DDS. Table S5. The association between absolute DDS change groups and CF in subgroups.
