# Supplementary material for: Dietary Diversity Changes and Cognitive Frailty in Chinese Older Adults: A Prospective Community-Based Cohort Study
Source: Nutrients. 2023 Aug 30;15(17):3784. doi: 10.3390/nu15173784 (PMC10490160; doi:10.3390/nu15173784)
Supplement: Supplementary file 1 [file nutrients-15-03784-s001.zip › nutrients-2567338-supplementary.pdf]

Supplementary Materials

Supplementary Methods

Definitions of DDS

Participants were categorised into 3 groups by their frequency of consumption of the 9 food: often ("almost every day"), occasionally ("once per week at least", "once per month at least", or "sometimes"), or rarely ("rarely or never") and each groups were scored as 2 (often), 1 (occasionally), and 0 (rarely) without taking a minimum intake into account. In general, we constructed overall DDS (including fresh vegetables, fruit, tea, garlic, beans, preserved vegetables, meat, fish, and eggs 9 food items) with the range of 0 to 18, constructed plant-based DDS (including fresh vegetables, fruit, tea, garlic, beans, and preserved vegetables 6 food items) with the range of 0 to 12, and constructed animal-based DDS (meat, fish, and eggs 3 food items) with the range of 0 to 6.

Items	Food items	Range
Overall DDS	fresh vegetables, fruit, tea, garlic, beans, preserved vegetables, meat, fish, and eggs	0 to 18
Plant-based DDS	fresh vegetables, fruit, tea, garlic, beans, preserved vegetables,	0 to 12
Animal-based DDS	meat, fish, and eggs	0 to 6

Definitions of 9 relative DDS change patterns

We categorized the overall DDS into 3 groups: high (13 to 18 score), medium (7 to 12 score), and low (0 to 6 score). Likewise, the plant-based DDS was categorized into high (9 to 12 score), medium (5 to 8 score), and low (0 to 4 score) groups, whereas the animal-based DDS was categorized into high (5 to 6 score), medium (3 to 4 score), and low (0 to 2 score) groups. We analyzed DDS at baseline, and 9 relative DDS change patterns based on the DDS at baseline and the first follow-up visit as follows: high-to-high, high-to-medium, high-to-low, medium-to-high, medium-to-medium, medium-to-low, low-to-high, low-to-medium, and low-to-low[1].

Items	Group (Score Range)	9 relative DDS change patterns
Overall DDS	High (13 to 18)	High-to-high, High-to-medium, High-to-low, Medium-to-high, Medium-to-medium, Medium-to-low, Low-to-high, Low-to-medium,
	Medium (7 to 12)	
	Low (0 to 6)	
Plant-based DDS	High (9 to 12)	
	Medium (5 to 8)	
	Low (0 to 4)	

Animal-based DDS	High (5 to 6)	Low-to-low.
	Medium (3 to 4)	
	Low (0 to 2)	

Definitions of 5 absolute DDS change patterns

We calculated the absolute change scores of the DDSs (DDS at first follow-up minus DDS at baseline) and categorized the absolute change patterns in overall DDS as follows: extreme decline (≤ -5 score), moderate decline (-4 to -2 score), stable (-1 to 1 score), moderate improvement (2 to 4 score), and extreme improvement (≥ 5 score)[2]. Similarly, the absolute change patterns in plant-based DDS were categorized into extreme decline (≤ -4 score), moderate decline (-3 to -2 score), stable (-1 to 1 score), moderate improvement (2 to 3 score), and extreme improvement (≥ 4 score); the absolute change patterns in animal-based DDS were categorized into extreme decline (≤ -3 score), moderate decline (-2 to -1 score), stable (0 score), moderate improvement (1 to 2 score), and extreme improvement (≥ 3 score).

Items	Group (Score Range)	5 relative DDS change patterns
Overall DDS	Extreme decline (≤ -5)	Extreme decline, Moderate decline, stable, Moderate improvement, Extreme improvement.
	Moderate decline (-4 to -2)	
	stable (-1 to 1)	
	Moderate improvement (2 to 4)	
	Extreme improvement (≥ 5)	
Plant-based DDS	Extreme decline (≤ -4)	
	Moderate decline (-3 to 2)	
	stable (-1 to 1)	
	Moderate improvement (2 to 3)	
	Extreme improvement (≥ 4)	
Animal-based DDS	Extreme decline (≤ -3)	
	Moderate decline (-2 to -1)	
	stable (0)	
	Moderate improvement (1 to 2)	
	Extreme improvement (≥ 3)	

Assessment of CF

Cognitive function was measured by the Chinese version of Mini-Mental State Examination (MMSE, score range 0 to 30) during each survey. Previous

investigations have shown MMSE validity and reliability[3]. It includes 24 items covering the test of orientation, registration, naming, attention, calculation, recall, and language. We defined cognitive impairment based on different education levels: < 18 for those with no formal education, < 21 for those with 1 - 6 years of education, and < 25 for those with more than 6 years of education[4]. Physical frailty was evaluated using the modified Fried criteria from self-report data, including weakness, exhaustion, shrinking, slowness, and inactivity[5, 6]. To be specific, weakness was defined by the participant failing to lift a bag weighing 5 kg. Exhaustion was defined by the participant answering “always”, “often”, or “sometimes” to either of the questions, “I felt old and useless”, or “I felt everything I did was an effort”. Shrinking was defined as BMI < 18.5 kg/m^2. Slowness was defined by the participant failing to walk for 1 km. Inactivity was defined as the absence (1 time per week or less) of the following activities: housework, outside activity, gardening, keeping a pet, livestock breeding, playing cards or moh-jong, and social activity. Physical frailty was defined as a participant meeting 3 or more criteria.

**Definition**	**Measured instruments**	**Items (Score Range)**
Cognitive impairment	Chinese version of Mini-Mental State Examination (MMSE, score range 0 to 30)	No formal education (< 18)
		1 - 6 years of education (< 21)
		More than 6 years of education (< 25)
Physical frailty	Modified Fried criteria (meeting 3 or more criteria)	Failing to lift a bag weighing 5 kg
		Feeling old, useless or everything was an effort
		Having BMI < 18.5 kg/m^2
		Failing to walk for 1 km
		Absence of the following activities (1 time per week or less): housework, outside activity, gardening, keeping a pet, livestock breeding, playing cards or moh-jong, and social activity.

Reference:

1. Liu D, Zhang XR, Li ZH, et al. Association of dietary diversity changes and mortality among older people: A prospective cohort study. Clin Nutr. May 2021;40(5):2620-2629. doi:10.1016/j.clnu.2021.04.012

2. Lv Y, Kraus VB, Gao X, et al. Higher dietary diversity scores and protein-rich food consumption were associated with lower risk of all-cause mortality in the oldest old. Clin Nutr. Jul 2020;39(7):2246-2254. doi:10.1016/j.clnu.2019.10.012

3. Zhang Z, Gu D, Hayward MD. Early life influences on cognitive impairment among oldest old Chinese. J Gerontol B Psychol Sci Soc Sci. Jan 2008;63(1):S25-33. doi:10.1093/geronb/63.1.s25

4. Zhang MY, Katzman R, Salmon D, et al. The prevalence of dementia and Alzheimer's disease

in Shanghai, China: impact of age, gender, and education. Ann Neurol. Apr 1990;27(4):428-37. doi:10.1002/ana.410270412
5. Fried LP, Tangen CM, Walston J, et al. Frailty in older adults: evidence for a phenotype. J Gerontol A Biol Sci Med Sci. Mar 2001;56(3):M146-56. doi:10.1093/gerona/56.3.m146
6. Wang HY, Lv X, Du J, Kong G, Zhang L. Age- and Gender-Specific Prevalence of Frailty and Its Outcomes in the Longevous Population: The Chinese Longitudinal Healthy Longevity Study. Front Med (Lausanne). 2021;8:719806. doi:10.3389/fmed.2021.719806

Supplementary Tables and Figures

Table S1. The numbers (percentages) of participants with missing covariates.

Table S2. Baseline characteristics of older adults according to absolute DDS change patterns.

Table S3. Sensitivity analyses of the association between DDS change patterns and all-cause mortality.

Table S4. The association between absolute DDS change groups and CF in plant-based and animal-based DDS.

Table S5. The association between absolute DDS change groups and CF in subgroups.

Figure S1. Flowchart of participant enrollment.

Table S1. The numbers (percentages) of participants with missing covariates.

Covariates	*n*	%
Living areas	1	0.01
Occupation	34	0.24
Sufficient income	2	0.01
Education levels	44	0.31
BMI	66	0.46
Hypertension	188	1.31
Diabetes	1	0.01
Stroke	1	0.01
Heart diseases	1	0.01

Table S2. Baseline characteristics of older adults according to absolute DDS change patterns.

Characteristics[a]	Total	DDS change patterns from baseline to first follow up				
		stable	Extreme decline	Moderate decline	moderate improvement	Extreme improvement
No. of participants	14382	4919	1791	3622	2931	1119
Age, mean (SD)	82.31 (10.83)	82.06 (10.84)	83.04 (11.16)	82.52 (10.83)	81.92 (10.70)	82.56 (10.54)
Sex						
Female	7729 (53.7)	2631 (53.5)	988 (55.2)	1940 (53.6)	1546 (52.7)	624 (55.8)
Male	6653 (46.3)	2288 (46.5)	803 (44.8)	1682 (46.4)	1385 (47.3)	495 (44.2)
Living areas						
Rural	8431 (58.6)	2914 (59.2)	1029 (57.5)	2119 (58.5)	1700 (58.0)	669 (59.8)
Urban	5951 (41.4)	2005 (40.8)	762 (42.5)	1503 (41.5)	1231 (42.0)	450 (40.2)
Marital statu						
Married	6086 (42.3)	2161 (43.9)	702 (39.2)	1544 (42.6)	1224 (41.8)	455 (40.7)
Not married	8296 (57.7)	2758 (56.1)	1089 (60.8)	2078 (57.4)	1707 (58.2)	664 (59.3)
Occupation						
Farmer	9111 (63.4)	3139 (63.8)	1109 (61.9)	2301 (63.5)	1849 (63.1)	713 (63.7)
Other	5271 (36.6)	1780 (36.2)	682 (38.1)	1321 (36.5)	1082 (36.9)	406 (36.3)
Education levels, y						
0	8271 (57.5)	2767 (56.3)	1040 (58.1)	2066 (57.0)	1682 (57.4)	716 (64.0)
≥1	6111 (42.5)	2152 (43.7)	751 (41.9)	1556 (43.0)	1249 (42.6)	403 (36.0)
Source of income						
Pension	2930 (20.4)	1024 (20.8)	358 (20.0)	740 (20.4)	597 (20.4)	211 (18.9)
Others	11452 (79.6)	3895 (79.2)	1433 (80.0)	2882 (79.6)	2334 (79.6)	908 (81.1)
Sufficient income						
Yes	11395 (79.2)	3886 (79.0)	1510 (84.3)	2978 (82.2)	2211 (75.4)	810 (72.4)
No	2987 (20.8)	1033 (21.0)	281 (15.7)	644 (17.8)	720 (24.6)	309 (27.6)

Living pattern						
Live alone/at nursing home	2533 (17.6)	853 (17.3)	317 (17.7)	580 (16.0)	556 (19.0)	227 (20.3)
Living with family member	11849 (82.4)	4066 (82.7)	1474 (82.3)	3042 (84.0)	2375 (81.0)	892 (79.7)
Smoking status						
Current smoker	3114 (21.7)	1047 (21.3)	384 (21.4)	798 (22.0)	647 (22.1)	238 (21.3)
Former smoker	2073 (14.4)	716 (14.6)	243 (13.6)	525 (14.5)	430 (14.7)	159 (14.2)
Nonsmoker	9195 (63.9)	3156 (64.2)	1164 (65.0)	2299 (63.5)	1854 (63.3)	722 (64.5)
Drinking status						
Current drinker	3229 (22.5)	1115 (22.7)	417 (23.3)	824 (22.7)	652 (22.2)	221 (19.7)
Former drinker	1551 (10.8)	501 (10.2)	189 (10.6)	373 (10.3)	349 (11.9)	139 (12.4)
Nondrinker	9602 (66.8)	3303 (67.1)	1185 (66.2)	2425 (67.0)	1930 (65.8)	759 (67.8)
Regular exercise	5215 (36.3)	1758 (35.7)	708 (39.5)	1359 (37.5)	1033 (35.2)	357 (31.9)
BMI, kg/m^2	20.23 (4.34)	20.30 (4.60)	20.31 (4.03)	20.24 (4.11)	20.11 (3.73)	20.05 (5.64)
Hypertension	6503 (45.2)	2143 (43.6)	834 (46.6)	1604 (44.3)	1366 (46.6)	556 (49.7)
Diabetes	322 (2.2)	115 (2.3)	45 (2.5)	84 (2.3)	61 (2.1)	17 (1.5)
Stroke	668 (4.6)	225 (4.6)	91 (5.1)	146 (4.0)	152 (5.2)	54 (4.8)
Heart diseases	1273 (8.9)	452 (9.2)	146 (8.2)	308 (8.5)	255 (8.7)	112 (10.0)

[a] Values represent the means ± SD or number (percentage). BMI: body mass index; DDS: dietary diversity score; SD: standard deviation.

Table S3. Sensitivity analyses of the association between DDS change patterns and CF.

Subgroups	DDS change patterns from baseline to first follow up								
	High-High	High-Medium	High-Low	Medium-High	Medium-Medium	Medium-Low	Low-High	Low-Medium	Low-Low
Overall DDS									
HR (95%CI)[a]	1.00 (reference)	1.30 (1.06, 1.59)	2.00 (1.49, 2.69)	0.98 (0.79, 1.23)	1.15 (0.95, 1.38)	1.77 (1.44, 2.17)	1.22 (0.78, 1.90)	1.45 (1.16, 1.81)	1.93 (1.50, 2.48)
HR (95%CI)[b]	1.00 (reference)	1.34 (1.10, 1.65)	1.97 (1.47, 2.65)	0.97 (0.78, 1.21)	1.23 (1.02, 1.48)	1.84 (1.50, 2.26)	1.16 (0.74, 1.82)	1.54 (1.24, 1.93)	1.97 (1.53, 2.53)
HR (95%CI)[c]	1.00 (reference)	1.30 (1.06, 1.59)	2.04 (1.52, 2.75)	0.99 (0.80, 1.24)	1.17 (0.97, 1.41)	1.79 (1.46, 2.20)	1.19 (0.76, 1.88)	1.49 (1.19, 1.86)	1.97 (1.53, 2.53)
HR (95%CI)[d]	1.00 (reference)	1.31 (1.06, 1.61)	2.08 (1.54, 2.80)	1.01 (0.81, 1.27)	1.19 (0.98, 1.44)	1.86 (1.51, 2.30)	1.27 (0.81, 2.00)	1.54 (1.23, 1.93)	2.04 (1.58, 2.63)
Plant-based DDS									
HR (95%CI)[a]	1.00 (reference)	1.49 (1.19, 1.87)	3.12 (2.37, 4.10)	1.26 (0.99, 1.60)	1.38 (1.12, 1.70)	2.10 (1.69, 2.62)	1.07 (0.70, 1.64)	1.74 (1.37, 2.20)	1.96 (1.53, 2.50)
HR (95%CI)[b]	1.00 (reference)	1.51 (1.20, 1.90)	3.17 (2.42, 4.17)	1.22 (0.95, 1.55)	1.40 (1.13, 1.73)	2.15 (1.72, 2.68)	1.01 (0.66, 1.55)	1.80 (1.42, 2.28)	2.00 (1.57, 2.56)
HR (95%CI)[c]	1.00 (reference)	1.45 (1.15, 1.83)	3.10 (2.36, 4.08)	1.26 (0.99, 1.60)	1.38 (1.12, 1.71)	2.09 (1.68, 2.61)	1.04 (0.67, 1.61)	1.75 (1.39, 2.22)	1.99 (1.56, 2.55)
HR (95%CI)[d]	1.00 (reference)	1.47 (1.17, 1.86)	3.18 (2.41, 4.18)	1.26 (0.99, 1.61)	1.40 (1.13, 1.73)	2.16 (1.73, 2.69)	1.13 (0.73, 1.73)	1.76 (1.39, 2.23)	2.05 (1.60, 2.63)
Animal-based DDS									
HR (95%CI)[a]	1.00 (reference)	1.14 (0.95, 1.36)	1.31 (1.03, 1.67)	0.93 (0.77, 1.13)	0.90 (0.76, 1.05)	1.26 (1.06, 1.51)	1.06 (0.80, 1.39)	0.98 (0.82, 1.18)	1.25 (1.03, 1.51)

HR (95%CI)[b]	1.00 (reference)	1.20 (1.00, 1.43)	1.31 (1.03, 1.66)	0.96 (0.79, 1.16)	1.00 (0.85, 1.18)	1.33 (1.12, 1.59)	1.03 (0.78, 1.36)	1.05 (0.88, 1.27)	1.25 (1.04, 1.52)
HR (95%CI)[c]	1.00 (reference)	1.11 (0.93, 1.33)	1.31 (1.03, 1.67)	0.93 (0.76, 1.12)	0.90 (0.76, 1.06)	1.26 (1.05, 1.51)	1.08 (0.82, 1.43)	0.98 (0.81, 1.18)	1.24 (1.02, 1.50)
HR (95%CI)[d]	1.00 (reference)	1.14 (0.95, 1.37)	1.33 (1.04, 1.70)	0.94 (0.77, 1.15)	0.92 (0.78, 1.09)	1.30 (1.08, 1.56)	1.10 (0.83, 1.46)	1.02 (0.84, 1.23)	1.30 (1.07, 1.58)

[a] Adjusting for the number of teeth (continuous variable) and the use of dentures (yes or no).

[b] Adjusting for the year of recruitment.

[c] Excluding participants with dementia at baseline.

[d] Excluding participants with missing covariates.

Table S4. The association between absolute DDS change groups and CF in plant-based and animal-based DDS.

Absolute DDS change patterns	Extreme decline	Moderate decline	Stable	Moderate improvement	Extreme improvement
Plant-based DDS	1.66 (1.49, 1.84)	1.17 (1.06, 1.28)	1.00 (reference)	1.06 (0.95, 1.19)	1.13 (0.97, 1.31)
Animal-based DDS	1.60 (1.31, 1.96)	1.31 (1.19, 1.44)	1.00 (reference)	1.05 (0.95, 1.17)	1.12 (0.86, 1.47)

Adjusted for age, sex, living areas, marital status, occupation, years of education, source of income, sufficient income, living pattern, smoking status, drinking status, regular exercise, BMI, hypertension, diabetes, stroke, and heart diseases.

Table S5. The association between absolute DDS change groups and CF in subgroups.

Subgroups	Extreme decline	Moderate decline	Stable	Moderate improvement	Extreme improvement	*P* for interaction
Age, years						0.435
<80	1.23 (0.89, 1.71)	1.04 (0.81, 1.34)	1.00 (reference)	0.84 (0.63, 1.11)	0.98 (0.65, 1.48)	
≥80	1.74 (1.55, 1.95)	1.14 (1.03, 1.26)	1.00 (reference)	0.99 (0.88, 1.11)	1.13 (0.96, 1.32)	
Sex						0.136
Male	1.82 (1.52, 2.20)	1.02 (0.86, 1.21)	1.00 (reference)	0.91 (0.75, 1.11)	1.07 (0.83, 1.38)	
Female	1.60 (1.40, 1.83)	1.19 (1.07, 1.34)	1.00 (reference)	1.02 (0.90, 1.16)	1.15 (0.96, 1.38)	
Living areas						0.911
Urban	1.62 (1.37, 1.91)	1.08 (0.94, 1.25)	1.00 (reference)	0.94 (0.80, 1.11)	1.13 (0.90, 1.41)	
Rural	1.71 (1.48, 1.97)	1.17 (1.03, 1.32)	1.00 (reference)	1.01 (0.88, 1.16)	1.11 (0.92, 1.35)	
Smoking						0.442
Never smoker	1.64 (1.45, 1.86)	1.14 (1.02, 1.27)	1.00 (reference)	1.02 (0.90, 1.15)	1.14 (0.96, 1.36)	
Current or former smoker	1.72 (1.40, 2.11)	1.10 (0.92, 1.33)	1.00 (reference)	0.87 (0.70, 1.08)	1.01 (0.75, 1.35)	
Drinking						0.770
Never drinker	1.58 (1.39, 1.80)	1.11 (1.00, 1.25)	1.00 (reference)	0.98 (0.86, 1.11)	1.16 (0.98, 1.38)	
Current or former drinker	1.90 (1.56, 2.32)	1.18 (0.99, 1.41)	1.00 (reference)	0.99 (0.81, 1.21)	1.02 (0.77, 1.35)	
Regular exercises						0.252
Yes	1.51 (1.25, 1.82)	1.01 (0.85, 1.21)	1.00 (reference)	0.82 (0.67, 1.00)	1.00 (0.75, 1.32)	
No	1.74 (1.53, 1.98)	1.17 (1.05, 1.31)	1.00 (reference)	1.06 (0.93, 1.20)	1.16 (0.97, 1.37)	

Adjusted for age, sex, living areas, marital status, occupation, years of education, source of income, sufficient income, living pattern, smoking status, drinking status, regular exercise, BMI, hypertension, diabetes, stroke, and heart diseases.

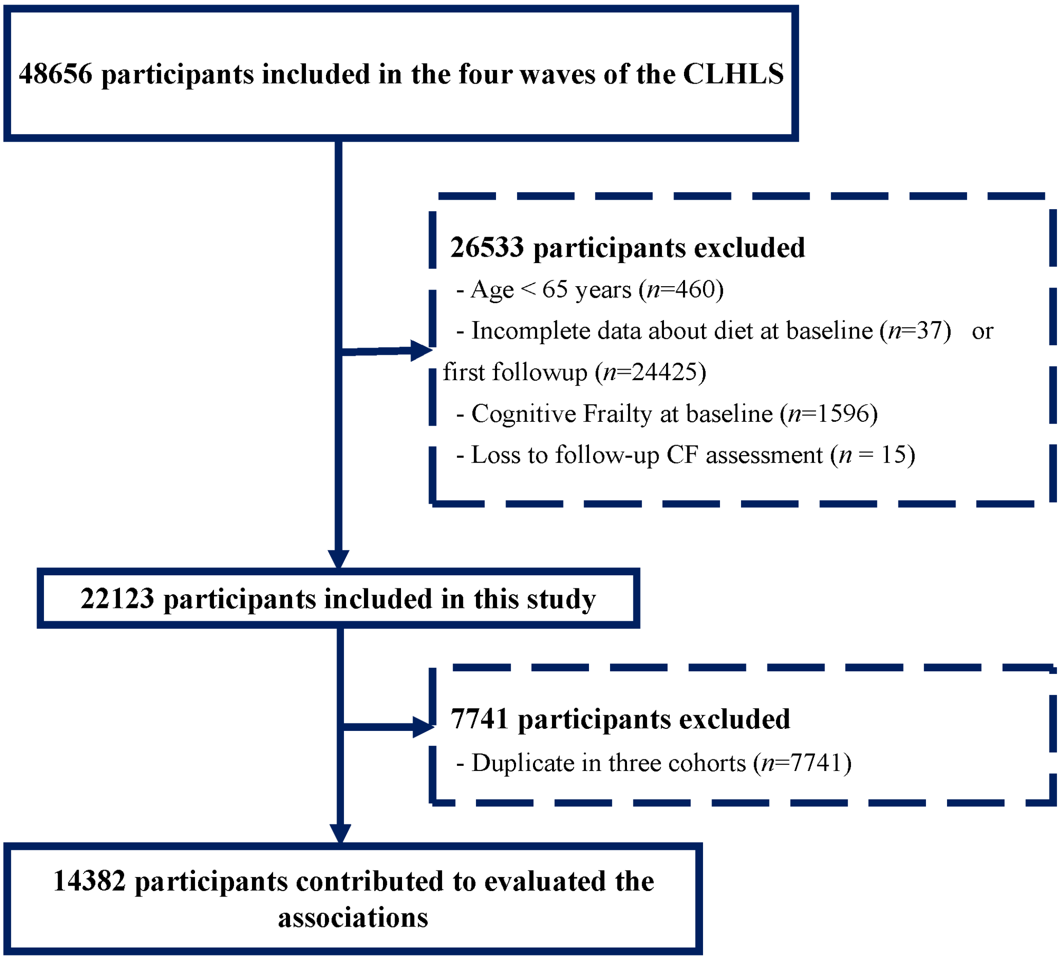

Figure S1. Flowchart of participant enrollment.
